# Parathyroid hormone related protein (PTHrP) in patients with pancreatic carcinoma and overt signs of disease progression and host tissue wasting

**DOI:** 10.1016/j.tranon.2023.101752

**Published:** 2023-08-02

**Authors:** Britt-Marie Iresjö, Serkan Kir, Kent Lundholm

**Affiliations:** a Surgical Metabolic Research Lab, Department of Surgery, Institute of Clinical Sciences, Sahlgrenska Academy, University of Gothenburg, Gothenburg 40530, Sweden; b Department of Cancer Biology, Dana-Farber Cancer Institute, Harvard Medical School, Boston, MA 02215, USA; c Department of Molecular Biology and Genetics, KoÇ University, Istanbul 34450, Turkey; d Department of Surgery, Sahlgrenska University Hospital, Region Västra Götaland, Gothenburg 41345, Sweden

**Keywords:** PTHrP, Cachexia, Pancreatic cancer, Oxidative metabolism

## Abstract

**Background:**

Cancer-cachexia is a complex syndrome secondary to physiological mechanisms related to classical hormone and immune alterations, where contributions of neuro-endocrine involvement have been less evaluated. Therefore, the aim of our study was to explore relationships between PTHrP and whole body metabolism in patients with progressive pancreatic carcinoma; relevant to “fat tissue browning”.

**Methods:**

Patient serum samples and clinical information were retrieved from earlier translational projects (1995-2005), at Sahlgrenska University Hospital in Gothenburg. Blood PTHrP levels were determined at Harvard medical School (2014). Patient data included: medical history, clinical laboratory tests, food diaries, resting metabolic expenditure, body composition, exercise capacity, Health-Related Quality of Life (SF-36) and mental disorders (HAD-scales).

**Results:**

Serum PTHrP was detectable in 17 % of all samples without significance to tumor stage. PTHrP-negativity at inclusion remained during follow-up. Mean PTHrP concentration was 262±274 pg/ml, without sex difference and elevation over time.

PTHrP-positive and negative patients experienced similar body weight loss (%) at inclusion, with a trend to deviate at follow ups (16.8±8.2% vs. 13.1±8.2%, p<0.06), where PTHrP concentrations showed correlations to weight loss, handgrip strength and Karnofsky performance, without difference in exercise capacity.

PTHrP-positivity was related to increased whole body fat oxidation (p<0.006-0.01) and reduced carbohydrate oxidation (p<0.01-0.03), independently of peripheral lipolysis. Metabolic alterations in PTHrP-positive patients were related to reduced Health Related Quality of life (SF: p<0.08, MH: p<0.02), and increased anxiety and depression (HAD 1-7: p<0.004; HAD 8-14: p<0.008).

**Conclusion:**

Serum PTHrP positivity in patients with pancreatic carcinoma was related to altered whole body oxidative metabolism; perhaps induced by “browning” of fat cells?

## Highlights

• Cancer-cachexia depends on mechanisms related to hormone and immune alterations.
• However, contributions by neuro-endocrine involvement have been overlooked.
• Our study implies that serum PTHrP is important in patients with pancreatic cancer.
• PTHrP protein may induce altered whole body oxidative metabolism and mood changes.
• However, the tissue origin of blood PTHrP remains to be clarified in cancer patients.

## Introduction

Clinical signs of cancer cachexia are obvious with excessive wasting of fat and muscle tissue at disease progression [1,2]; in parts explained by local and systemic inflammation [3,4], initial loss of adequate appetite [5], elevated energy expenditure [6], hormone alteration [7,8] and later on severe anorexia [9]. The search for factors explaining the mechanism(s) of disrupted homeostasis in cancer cachexia has been ongoing for decades [10], with expectation that cancer cachexia is different from activation of normal physiological mechanisms during trauma, infection, and neuro-endocrine stress reactions. However, effector mechanisms may be quantitatively different in various patients, probably dependent on the magnitude of various afferent signals, related to tumor immunity and metabolomics. Therefore, it is difficult to define practically useful biomarkers to either predict or monitor disease progression and outcomes beyond traditional tissue-related biomarkers in oncology; markers that are usually not related to effector mechanisms in host organ functions. Thus, a revival of a possibility related to Parathyroid hormone related protein (PTHrP) was recently presented by Kir et al. [11], demonstrating PTHrP dependent mechanisms related to adipose tissue browning and thermogenesis [11], possibly important for fat depletion in cancer cachexia [12,13].

Involvement of PTHrP behind wasting mechanisms in tumour disease was first suggested from rodent cachexia models, where blocking tumor-secreted PTHrP with antibodies alleviated cachexia [14,15] suggesting attenuation of effector mechanisms to prevent tumor-host wasting. In addition, anorexogenic properties of PTHrP have been proposed [16]. However, neuro-endocrine mechanisms on energy homeostasis in small animal models, highly dependent on extremely high metabolic rate per body mass, may be less relevant compared to a majority of slowly growing human tumors. Some evidence for PTHrP involvement in human cancer cachexia has been suggested [11,[17], [18], [19]]. Also, it was reported that a subset of human pancreatic carcinomas showed copy number gains of PTHrP (encoded by PTHLH gene), as part of KRAS amplification, which is frequent in pancreatic carcinomas [20]. Therefore, we deemed it relevant to re-examine the appearance and presence of blood PTHrP in patients with pancreatic carcinoma and overt signs of progressive disease and wasting. Pancreatic carcinoma in human is a main and consistent kind of cancer-induced wasting syndrome with involvement of anorexia, pain, endocrine disorders as well as gastro-intestinal functional disorders.

## Material and methods

### Patient cohort

Patient samples were collected in connection with various translational projects (years 1995-2005) in a bio-bank at Department of Surgery, Sahlgrenska University Hospital in Gothenburg, at recommended fasting conditions [3,7,[21], [22], [23]]. All patients participated in research programs to investigate metabolic, nutritional and functional profiles related to cancer cachexia. [3,4,7,21,23]. All participants gave informed consent and all study protocols have been approved by the Regional Board of Ethics in Gothenburg (288-93, S-141-02).

Blood-plasma and serum samples were stored at -80°C until analyses, which were usually performed within 1-2 months after collection. Serum PTHrP levels were later on (2014) analyzed by a commercially available PTHrP(1-34) ELISA assay (S-1227, Bachem), at the Department of cancer biology, Harvard medical school. Assay detection limit was 10 pg/ml. In total 128 samples were available from 94 patients with pancreatic cancer (stage III or IV); and 34 samples from 11/94 patients were collected during follow-up visits 2-36 months from “inclusions”. All patients had advanced tumor stage assessed either at surgical explorations or by diagnostic imaging (29 patients with liver metastases; 54 loco-regional spread; 4 lung- and peritoneal metastases; 7 multiple metastases). Clinical characteristics at inclusion investigations, before any disease treatment, are presented in Table 1.

**Table 1:** Clinical characteristics of 94 patients at study inclusion.

**Variable**	**Mean±SD (n)**
***Patient caracteristics***	
Male/female (n)	51/43 (94)
Age, years	69±11 (94)
Blood pressure, mmHg	140±21 (94)
Pulse rate, beats/min	75±10 (68)
**Body temp, °C**	36.8±0.5 (69)
**BMI, kg/m^2^**	22.1±3.5 (93)
Weight, kg	64.9±13.3 (93)
Weight loss, %	13.0±8.2 (91)
***Blood tests***	
Hemoglobin, g/L	119±16 (94)
Albumin, g/L	33.6±5.3 (93)
Blood glucose, mmol/L	7.4±3.2 (79)
Glycerol, µmol/L	51±32 (93)
FFA, mmol/L	0.58±0.22 (22)
Triglycerides, mmol/L	1.0±0.4 (23)
IGF-1, µU/L	105±65 (88)
Insulin, U/L	13±16 (39)
Leptin, µg/L	4±2 (19)
C-reactive protein, mg/L	32±41 (92)
**White blood cell count, 10^9^/L**	7.8±2.7 (94)
Sodium, mmol/L	138±3 (93)
Potassium, mmol/L	4.1±0.5 (93)
Total calcium, mmol/L	2.3±0.1 (93)
Creatinine, µmol/L	88±23 (93)
Bilirubin, µmol/L	16±19 (93)
ASAT, µkat/L	0.6±0.5 (93)
ALAT, µkat/L	0.8±0.6 (93)
***Food intake & metabolism***	
REE, kcal/kg/day	22.9±3.6 (90)
Food intake, kcal/day	1639±539 (79)
Energy balance, kcal/day	161±500 (78) **^a^**
***Body composition by DEXA***	
Whole body fat, kg	15.3±8.5 (84)
Lean body mass, kg	46.3±9.8 (84)
Bone mineral content, kg	2.6±0.7 (84)
Bone mineral density, g/cm^2^	1.12±0.15 (84)

a: significantly different from zero or negative energy balance.

REE: Resting energy expenditure

### Clinical and laboratory variables

Records of patient data including sex, age, medical history and clinical laboratory tests as well as additional registrations of body composition by DEXA, exercise capacity on a treadmill, Health-Related Quality of Life questionnaire (SF-36), mental disorders (HAD-scales) and food diaries were retrieved from our database, according to previous publications [7,[21], [22], [23]]; and analyzed in comparison to serum levels of PTHrP. All parameters were not available from all individuals as indicated in Table 1. Net lipolytic ratio were estimated as venous plasma glycerol divided by kg whole body fat by DEXA, given that visceral clearance of glycerol was comparable between PTHrP positive and negative patients [24,25]. Whole body fat and carbohydrate oxidative metabolism was estimated by standard indirect calorimetry [5]. Energy balance were calculated as intake of kcal (food diaries) - measured resting energy expenditure (REE) by indirect calorimetry.

### Statistical analyses

**Fig. 1:**
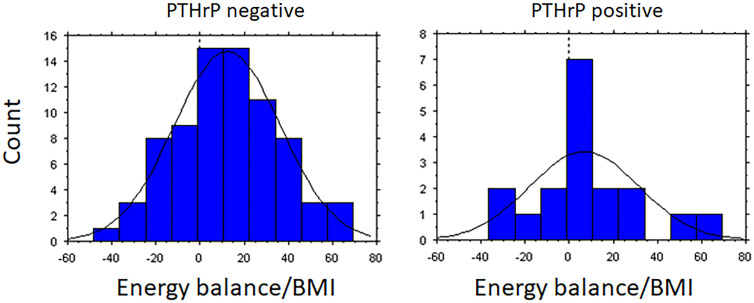
Distribution of daily energy balance (kcal/BMI/day) at study inclusion and follow up investigations in PTHrP positive and PTHrP negative patients. PTHrP-negative, n=78. PTHrP-positive, n=18.

Results are presented as mean ±SD (t-test statistics), since graphic evaluations supported normal distributions among study and control patients (Fig. 1). One sample t-test was used to confirm any significant difference from zero. However, differences between groups were analyzed by one way ANOVA, which rather depends on equal distribution (variance) of variables than normal distribution among study and control patients (Table 2). Statistical evaluation was performed on log transformed values when variables are evidently not normally distributed, although continuous such as survival (days). Applied chi-square is based on data ranking (nominal scale). p<0.05 was regarded statistically significant in two-sided tests. Statistical trends are indicated in tables with p<0.10. Multiple- and univariate standard regressions were used to explore possible interactions between PTHrP and various patient variables (Tables 3, 4). All analyses were performed in Statwiev for windows v.5.0.1.

**Table 2:** Selected clinical characteristics of PTHrP-negative and PTHrP-positive patients at study inclusion. Data are presented as mean ±SD (n).

**Variable**	**PTHrP-negative (78)**	**PTHrP-positive (16)**	P < 0.05 *
***Patient characteristics***			
Male/female (n)	41/37	10/6	
Age, years	70±11 (78)	67± 10 (16)	
Weight, kg	64.7±13.6 (77)	68.0±15.7 (16)	
**BMI, kg/m^2^**	22.1±3.4 (77)	22.2±3.9 (16)	
Weight loss, %	12.6±8.2 (75)	15.1±8.1 (16)	
Survival days, *	136; 49,379 (78) *	69; 16,291 (16) *	
***Blood tests***			
Total calcium, mmol/L	2.31±0.12 (77)	2.25±0.10 (16)	0.05
C-reactive Protein, mg/L	31±39 (77)	39±48 (16)	
Albumin, g/L	33.8±5.1 (76)	32.1±5.3 (16)	
Bilirubin, µmol/L	16±20 (77)	19±16 (16)	
White blood cell count,	7,67±2.6 (76)	8.2±3.3 (16)	
***Whole body metabolism***			
REE, kcal/day	1467±272 (75)	1542±317 (16)	
REE, kcal/kg/day	22.9±3.4 (74)	23,0±4.0 (16)	
REE/BMI	67±11 (74)	71±14 (16)	
RQ	0.79±0.07 (74)	0.75±0.05 (16)	0.01
Fat oxidation, g/min/kg	1.46±0.57 (74)	1.85±0.55 (16)	0.01
Fat oxidation, g/min/BMI	4.3±1.7 (74)	5.6±1.7 (16)	0.006
Carbohydrate oxidation, g/min/kg	1.4±1.3 (74)	0.6±0.89 (16)	0.01
Carbohydrate oxidation, g/min/BMI	4,1±3,9 (74)	1,75±2,8 (16)	0.03
***Food intake***			
Total intake, kcal/day	1643±546 (65)	1621±523 (14)	
Total intake, kcal/kg/day	26.1±9.3 (65)	24.0±5.7 (14)	
Total intake, kcal/BMI/day	76±28 (65)	73±6 (14)	
Protein, g/day	67±22 (65)	58±21 (14)	
Fat, g/day	68±27 (65)	67±35 (14)	
Carbohydrates, g/day	187±63 (65)	173±54 (14)	
Energy balance, kcal/kg/day	3.1±8.0 (63)	1.21±5.32 (14)	
Energy balance, kcal/BMI/day	8.84±23.6(63)	4.15±15.1 (14)	
***Body composition (DEXA)***			
Whole body fat, kg	15.1±8.27 (72)	17.1±10.0 (16)	
Lean body mass, kg	46.2±9.9.5 (72)	47.3±11.6(16)	
Bone mineral content, kg	2.50±0.62 (72)	2.84±0.89 (16)	
**Bone mineral density, (g/cm^2^)**	1.11±0.14 (72)	1.17±0.16 (16)	

RQ= whole body respiratory quotient.

* Statistical analysis were performed on log transformed values (mean days;-1SD,+1SD).

**Table 3:** Relationship between PTHrP concentrations and weight loss and body functions at study inclusion.

**Variable (n)**	**Regression**	**r-value**	**P<value**
Body weight loss, % (16)	y= 17.8x-46	0.49	0.05
Handgrip strength (15)	y=564 -13.8x	-0,48	0.06
Karnofsky performance status (15)	y=1398 -15.5x	-0.56	0.03

**Table 4:** Multiple regression analysis with weight loss (%) as dependent factor. All patients at inclusion and follow up (n=100).

**Independents**	**Coeffficient**	**Standard error**	**Standardized coefficient**	**t-value**	**p-value**
Intercept	13.184	7.983	13.184	1.652	0.10
Fat oxidation/BMI	1.310	0.694	0.312	1.88	0.06
Carbohydrate oxidation/BMI	0.492	0.296	0.251	1.661	0.10
Net lipolytic ratio	0.171	0.122	0.14	1.408	0.16
PTHrP (pg/ml)	0.013	0.007	0.178	1.806	0.07
Albumin (g/L)	-0.258	0.166	-0.160	-1.560	0.12
White blood cells/L	-0.075	0.353	-0.23	-0.212	0.83

The regression model was significant at p<0.01, R=0.40

## Results

Serum PTHrP was detectable in 17 % of all samples (22 of 128), without any statistically significant relationship to clinical tumor stage. All patients, who were PTHrP-negative at inclusion remained serum negative during follow-up periods. Patient characteristics in PTHrP-positive and PTHrP-negative patients are presented in Table 2. The number of positive serum samples was significantly lower compared to by chance observations (p<0.01). There was no sex-difference among serum- positive and negative blood samples. Mean PTHrP concentration was 262±274 pg/ml (SD; minimum 31, maximum 1122) among positive patients. Sixteen positive patients were identified at inclusion and additional six positive samples were obtained at follow-up visits; found in two male patients, without any significant increase over time (2-36 months, p=0.3).

On a group level, patients were in estimated positive energy balance at the occasion of “inclusion investigations” and clinical assessments despite advanced disease stages (Table 1), but the distributions of individual energy balances across the range of negative to positive energy balance were large (Fig. 1). However, mean daily energy balance was significantly positive in PTHrP-negative patients (9±3 Kcal/BMI; p<004). and not different from zero balance in PTHrP positive patients (4±1 kcal/BMI; p>0.05).

PTHrP-positive patients had significantly increased whole body fat oxidation (either normalized to body mass index (BMI) or to kg body weight) and significantly reduced whole body carbohydrate oxidation; as indicated by a significantly reduced respiratory quotient (RQ) in serum positive PTHrP-patients; a variable that is independent of any normalization to body weight or BMI (Table 2).

PTHrP-positive patients indicated similar body weight loss (%) as PTHrP-negative patients at inclusion (Table 2), with a trend to deviate later on at follow ups (16.8±8.2% vs. 13.1±8.2%, p<0.06). Serum PTHrP concentrations correlated to relative weight loss (r=0.49, p<0.05), Handgrip strength (r=-0.48, p<0.06) and Karnofsky performance status (-0.56, p<0.03) at inclusion (Table 3). Multiple regression analyses on all patients supported that whole body fat oxidation and PTHrP-concentrations were most related to weight loss (p<0.06-0.07), compared to carbohydrate oxidation, lipolysis (p<0.1-0.16), and systemic inflammation (albumin p<0.12; LPK<0.83) (Table 4).

Overall, observed metabolic alterations on whole body metabolism were significantly related to reduced Health Related Quality of life when split by PTHrP positivity versus PTHrP-negativity in patients, evaluated by SF-36 (SF: p<0.08, MH: p<0.02) as well as increased anxiety and depression evaluated by HAD-scales (HAD 1-7: p<0,004; HAD 8-14: p<0.008), analyzed on all available serum samples at inclusion and follow ups.

## Discussion

Cancer cachexia is a well-recognized phenomenon in patients with malignant disease, secondary to both disease progression and various treatment options as radiation- and chemotherapy [10]. Pure disease relationships behind tumour-host reactions have been the focus of many research investigations during decades with an expectation to explain the mechanisms behind and sometimes to find a “magic bullet” to prevent or at least attenuate cachexia development in patients on palliative regimens. In large, it is evident that cancer cachexia is a state of complex interactions between neuro-endocrine, classic hormones and substrate alterations in response to cell and tissue damage with secondary activation of inflammation and immune cell activities [26]. Accordingly, palliative means have to rely on multifactorial interventions and supportive regimens as nutrition, pain-relief, and attenuation of elevated metabolic signals [7,8,21]. So far, several factors that reflect immune activation and systemic inflammation have been most rewarded as markers of disease activities [9]. Recently, Professor Spiegelman and colleagues presented an interesting observation with the potential importance of PTHrP, to induce adipose tissue browning to explain fat loss and subsequent cachectic events, in tumor-bearing mice; a model with several convincing observations to imply possible important implications for clinical cancer [11]. This publication represents a revival of interest behind potential importance of neuro-endocrine mechanisms to explain cachexia, although earlier clinical attempts to reveal similar findings in clinical materials had been less rewarding [17]. Therefore, we (B-MI; KL) decided to propose our possibility to contribute to further knowledge on PTHrP in patients with progressive pancreatic carcinoma; a group of cancer patients with well-recognized and obligatory development of cachexia. Accordingly, we found it interesting to retrospectively analyze bio-banked serum samples from pancreatic cancer patients, who had participated in our clinical evaluations of metabolic, nutritional and self-recorded Health Related Quality of Life [3,22,23]. The initial and principal investigations were conducted when patients appeared or were referred to our hospital unit for considerations of surgical treatments. At this time most patients were usually untreated, with exceptions of conventional pain treatment and sleeping pills when needed; and no patient had received chemotherapy or any specific palliative treatment before our “inclusion investigations”.

The metabolic and clinical characteristics show a group of patients that are characterized by overt and progressive cachexia; i.e. weight loss (13%), anemia (119g/L), fatigue with signs of depression and anxiety, but minimal alterations of liver function tests with normal bilirubin concentrations on a group level; although few patients had slightly elevated serum bilirubin. This is an important requirement since major liver abnormality makes metabolic alterations difficult to interpret in the context of neuro-endocrine alterations. Likewise, blood total calcium levels were within the normal ranges, in both PTHrP positive and negative patients.

The distribution of daily food intake and estimated energy balance among our patients is interesting to consider [5]. It is likely that small alterations in appetite can induce significant alterations in body compositions over time, although loss of appetite may either have metabolic, endocrine, toxic or even mechanical and a sensational influence on the gastro-intestinal tract. Therefore, it was interesting that the mean daily energy intake (kcal/kg) was almost similar among PTHrP-positive and negative patients, while derived daily energy balance was significantly positive in PTHrP-negative patients (9±3 Kcal/BMI; p<004) and not different from zero balance in PTHrP-positive patients (4±1Kcal/BMI) (Fig. 1). This small dissonance between PTHrP-positive and negative patients may be a trigger of increased fat oxidation compared to decreased carbohydrate oxidation; (Tables 2, 4). A metabolic switch towards increased fat oxidation could well explain weight loss around 2 kg per 6 months in a majority of patients compared to a condition with balanced whole body substrate oxidation.

It is likely that significant alterations are consistent, among PTHrP-positive and negative patients at “inclusion” and across the entire investigative periods (0-32 month), although interpretations of complex interactions among physiologic and disease related adaptations must be careful in the present kind of studies. Thus, effects by unavoidable palliative support factors (insulin, pain relief, supportive extra caloric intake etc.) cannot be excluded, as an unavoidable part of dignified medical care of patients without curable hope. Despite such methodological limitations in clinical studies, it is evident that distinct and consistent observations remain. Seen together, present metabolic observations are compatible with, insulin resistance, increased adrenergic activities, systemic inflammation, which all may be primary effector mechanisms to induce fat browning [27].

Described altered whole body oxidative metabolism may well be secondary to primary appetite loss, without influence by adrenergic mechanisms but related to insulin resistance to mobilize free fatty acids from fat stores to support heart and brain tissues, as the increasingly preferred oxidative substrate in conditions with appetite loss and later on anorexia. However, multiple regression analysis implied that peripheral induction of lipolysis (“net lipolytic ratio”) was not a significant factor to predict fat metabolism among serum PTHrP-positive and negative patients (Table 4). Therefore, it is more likely that a change in whole body oxidative substrate metabolism in pancreatic carcinoma, for increased fat utilization, was less dependent on peripheral lipolysis, although present methodology may be too insensitive to clearly clarify mechanisms.

The observation that none of PTHrP-negative patients became PTHrP-positive within 32 months observations, despite disease progressions and death, may suggest that PTHrP in pancreatic carcinoma is not a progressively inducible “tumor factor” [20,28]. It remains to be evaluated whether blood PTHrP originates from tumor-cells or any other untransformed normal tissue(s). PTHrP is normally expressed in most tissues, including brain, with calcium sensing among its physiological roles [29,30]. Thus, a speculation may be that the subtle alterations in mental disorders related to reduced self-recorded quality of life, including anxiety and signs of mood depression in PTHrP positive patients may be effects related to hypothalamic neuronal signaling.

In conclusion, the present observations show that neuroendocrine alterations are probably significant for development of metabolic and functional alterations in patients with pancreatic carcinoma and perhaps also in other types of solid tissue malignancy. Our data may also support the view that fat browning is a significant mechanism behind fat depletion in sub-groups of clinical cancer. Thus, it remains to apply investigative procedures to confirm such a definite statement in patients who are serum PTHrP positive [13,31].

## Funding

The study was supported by grants from the Swedish Cancer Society. The funder had no role in study design, data collection and analysis, decision to publish, or preparation of the manuscript.

## CRediT authorship contribution statement

**Britt-Marie Iresjö:** Data curation, Formal analysis, Writing – original draft, Writing – review & editing. **Serkan Kir:** Investigation, Writing – review & editing. **Kent Lundholm:** Conceptualization, Funding acquisition, Formal analysis, Writing – original draft, Writing – review & editing.

## Declaration of Competing Interest

The authors declare that they have no known competing financial interests or personal relationships that could have appeared to influence the work reported in this paper.
